# Interactive virtual reality assessment of aggressive social information processing in boys with behaviour problems: A pilot study

**DOI:** 10.1002/cpp.2620

**Published:** 2021-06-17

**Authors:** Rogier E. J. Verhoef, Anouk van Dijk, Esmée E. Verhulp, Bram O. de Castro

**Affiliations:** ^1^ Department of Developmental Psychology Utrecht University Utrecht The Netherlands; ^2^ Research Institute of Child Development and Education University of Amsterdam Amsterdam The Netherlands; ^3^ Centre for Urban Mental Health University of Amsterdam Amsterdam The Netherlands

**Keywords:** aggression, behaviour problems, children, pilot study, social information processing, virtual reality

## Abstract

Children's aggressive behaviour is partly determined by how they process social information (e.g., making hostile interpretations or aiming to seek revenge). Such aggressive social information processing (SIP) may be most evident if children are emotionally engaged in actual social interactions. Current methods to assess aggressive SIP, however, often ask children to reflect on hypothetical vignettes. This pilot study therefore examined a new method that actually involves children in emotionally engaging social interactions: interactive virtual reality (VR). We developed a virtual classroom where children could play games with virtual peers. A sample of boys (*N* = 32; ages 8–13) from regular and special education reported on their SIP in distinct VR contexts (i.e., neutral, instrumental gain and provocation). They also completed a standard vignette‐based assessment of SIP. Results demonstrated good convergent validity of interactive VR assessment of SIP, as indicated by significant moderate to large correlations of VR‐assessed SIP with vignette‐assessed SIP for all SIP variables except anger. Interactive VR showed improved measurement sensitivity (i.e., larger variances in SIP compared to vignettes) for aggressive responding, but not for other SIP variables. Discriminant validity (i.e., distinct SIP patterns across contexts) of interactive VR was supported for provocation contexts, but not for instrumental gain contexts. Last, children were more enthusiastic about the VR assessment compared to the vignette‐based assessment. These findings suggest that interactive VR may be a promising tool, allowing for the assessment of children's aggressive SIP in standardized yet emotionally engaging social interactions.

**Key Practitioner Message**

- Interactive VR is a promising method to assess children's aggressive social information processing.
- Interactive VR showed to be more sensitive in assessing individual differences in children's aggressive responding than a standard vignette assessment.
- Children were more enthusiastic about the interactive VR assessment of their social information processing than about a standard vignette assessment.

## 1 INTRODUCTION

Children frequently encounter challenging social situations such as being laughed at, losing a game or being excluded. How children mentally process such situations influences their subsequent behaviour (Anderson & Bushman, 2002; Crick & Dodge, 1994; Lemerise & Arsenio, 2000). The social information processing (SIP) model distinguishes several internal processing steps children engage in before responding to social events: (1) encoding and (2) interpreting social cues, (3) setting interactional goals, (4) generating and (5) evaluating responses and (6) enacting a selected response (Crick & Dodge, 1994). Over the past decades, this SIP model has been shown to provide a convincing theoretical framework for the understanding, prevention and treatment of aggressive behaviour problems (for a review, see De Castro & Van Dijk, 2017). Children's aggression has been shown to derive from deviations in each of these SIP steps, such as perceiving more threatening cues, attributing more hostile intentions to others, pursuing revenge or instrumental goals more often, generating more aggressive responses and evaluating aggressive responses more positively (for reviews, see De Castro & Van Dijk, 2017; Dodge, 2011). Moreover, intervention studies have shown that changing children's SIP can reduce aggression (Lochman et al., 2017; Lochman et al., 2019; Maixner‐Schindel & Shechtman, 2021; Wilson & Lipsey, 2006). Given the important role of SIP underlying children's aggression, valid assessment of SIP is essential. The present pilot study examines a new method to assess children's SIP in an ecologically valid manner: interactive virtual reality (VR).

Current methods to assess aggressive SIP have important shortcomings. Until now, most studies have assessed children's SIP using hypothetical stories, where children are asked to imagine that a hypothetical social event is actually happening to them and to reflect on their SIP in response to this hypothetical event. Using such hypothetical vignettes limits the ecological validity of SIP assessment, especially because many children may only show aggressive SIP when they are emotionally engaged in actual social events (Anderson & Bushman, 2002). Strong emotions such as anger, embarrassment or excitement may trigger aggressive cognitions that would not be triggered when children feel calm (Lemerise & Arsenio, 2000). For example, children may only attribute hostile intent to others when they feel frustrated or may only pursue instrumental goals when they strongly desire an object. The relevance of assessing children's aggressive SIP in emotionally engaging social situations is emphasized by empirical work showing that inducing negative emotions elicits more aggressive SIP and behaviour (e.g., Caporaso & Marcovitch, 2021; De Castro et al., 2003; Reijntjes et al., 2011). Thus, an ecologically valid assessment of children's aggressive SIP requires the use of emotional engaging social situations.

A few earlier attempts to promote ecological validity have used staged real‐time conflicts with (alleged) peers or child actors (Hubbard et al., 2001; Kempes et al., 2008; Steinberg & Dodge, 1983; Van Dijk et al., 2019). A meta‐analysis has demonstrated such studies found stronger associations between hostile intent attribution and aggression (*d* = 1.33) than studies using vignettes (*d* = 0.23 to 0.44; Verhoef et al., 2019). This suggests that ecologically valid methods may improve the assessment of children's aggressive SIP. Research using of staged conflicts, however, can be ethically challenging and difficult to standardize. First, staged conflicts are prone to escalation, complicating adherence to ethical guidelines. Second, when staging real‐time conflicts between children, it is difficult to ensure that child actors behave identically with each participant, limiting standardization. As such, there is a need for innovative methods to assess children's aggressive SIP that can combine highly emotionally engaging, realistic social interactions with adequate standardization and adherence to ethical guidelines.

Interactive VR may provide a viable solution for limitations encountered by previous research. VR technology is already used for the assessment and treatment of various forms of psychopathology in adults (for reviews, see Carl et al., 2019; Emmelkamp & Meyerbröker, 2021; Freeman et al., 2017). For children, though, research using VR is relatively limited. VR has been utilized for the treatment of autism and attention deficit‐hyperactivity disorder and for teaching emotion regulation skills to prevent risk taking behaviour in adolescents (Hadley et al., 2019; Mesa‐Gresa et al., 2018; Shema‐Shiratzky et al., 2018). In addition, one study assessing SIP in children with autism spectrum disorder has used non‐interactive VR, in which children navigated an avatar through a simulated 3D environment by selecting response options using a computer menu (Russo‐Ponsaran et al., 2018). This method, however, may be less suitable to assess children's aggressive SIP because not being able to respond through actual behaviour in the VR environment may lower children's emotional engagement. To our knowledge, interactive VR has not been previously used to assess children's aggressive SIP.

Interactive VR may have several benefits for the assessment of children's aggressive SIP. First, it enhances ecological validity by immersing children in an emotionally engaging environment where they can interact with, and possibly aggress against, virtual peers. Second, interactive VR allows for rigorous experimental control. By controlling the course and content of social events in VR, researchers can standardize scenarios between participants and adhere to ethical guidelines. Third, VR can be flexibly used to present children with various different contexts, enabling researchers to assess individual differences in aggressive behaviour and associated SIP patterns. For the present study, we developed an interactive VR environment, aiming to optimize these benefits to provide an ecological valid assessment of children's aggressive SIP. In this first pilot study, we targeted school‐aged boys with different levels of behaviour problems to maximize potential variance stemming from differences in aggression. As such, we could examine whether our interactive VR would be a valid assessment method for boys across the whole spectrum from non‐aggressive children to children with severe aggressive behaviour problems.

**FIGURE 1:**
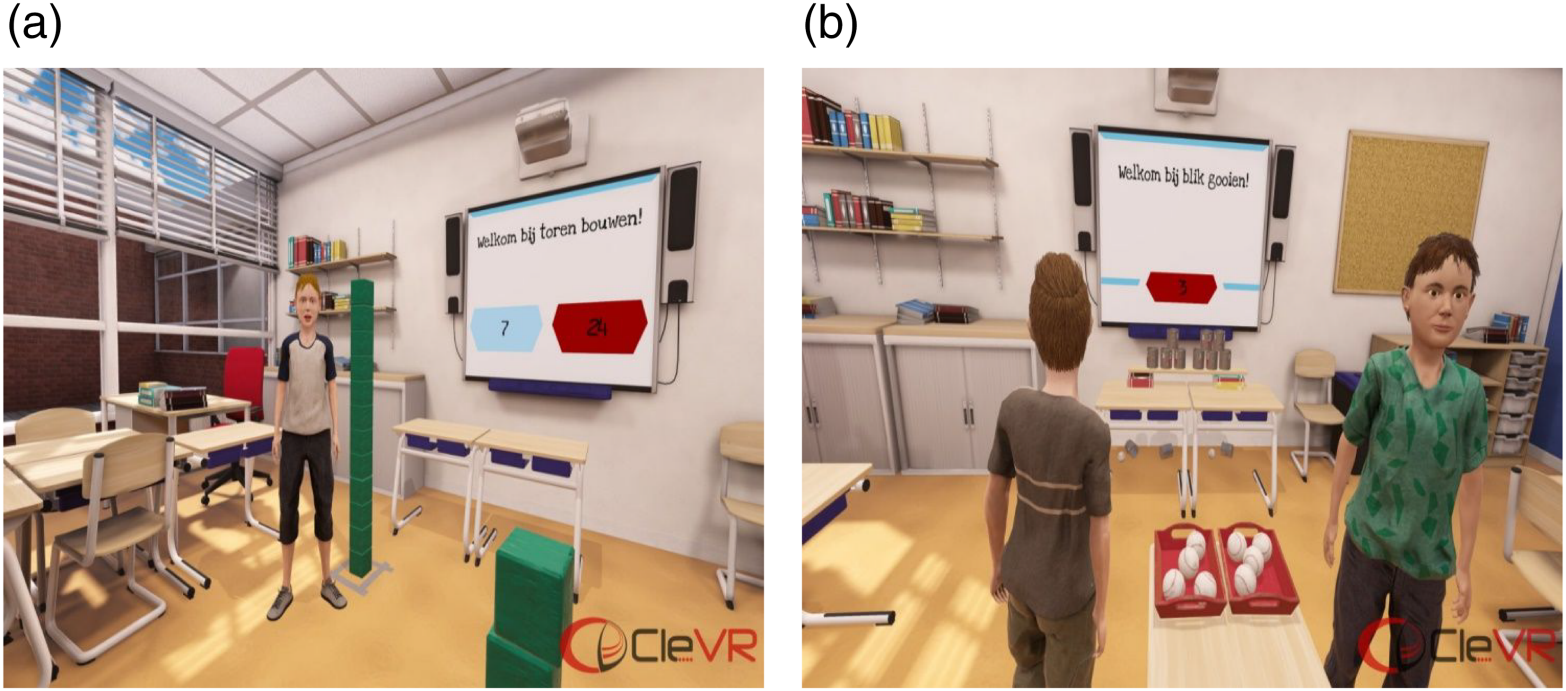
(a and b) Virtual classroom with the tower game or with the cans game [Colour figure can be viewed at wileyonlinelibrary.com]

First, to promote ecological validity, we designed the VR environment to be interactive and realistic. Participants are visually completely immersed in a virtual classroom that, just like the real world, responds naturally to each single motion (Figure 1). Participants can freely walk around in the virtual classroom (in reality, they walk around in a demarcated space in an empty room at their school with the VR glasses on). They interact with virtual peers in similar fashion as in real life: through verbal and physical behaviours. They use controllers that mimic their hands in VR, allowing them to use objects and play games. The virtual peers are manually controlled by the experimenter using standardized speech options and physical actions. This VR environment allows for various engaging interactions to assess children's SIP, such as building a 2‐m‐high block tower that is being bumped over by a virtual peer (i.e., an ambiguous provocation).

A particularly sensitive aspect of ecological validity is the assessment of participants' aggressive behaviour in the VR environment. Aggressive behaviour is defined as ‘any behavior directed towards another individual with the intent to cause harm’ (Anderson & Bushman, 2002, p. 27). Thus, to ensure ecological validity, it is important that children believe that their aggressive behaviour in VR does actual harm to the virtual peer. This is not self‐evident, as many children play digital games where they use violence against characters they know do not exist. Therefore, we presented our virtual classroom as an actual classroom where participants allegedly met with real children from other schools who also participated in our study and were simultaneously logged on to the VR environment.

**FIGURE 2:**
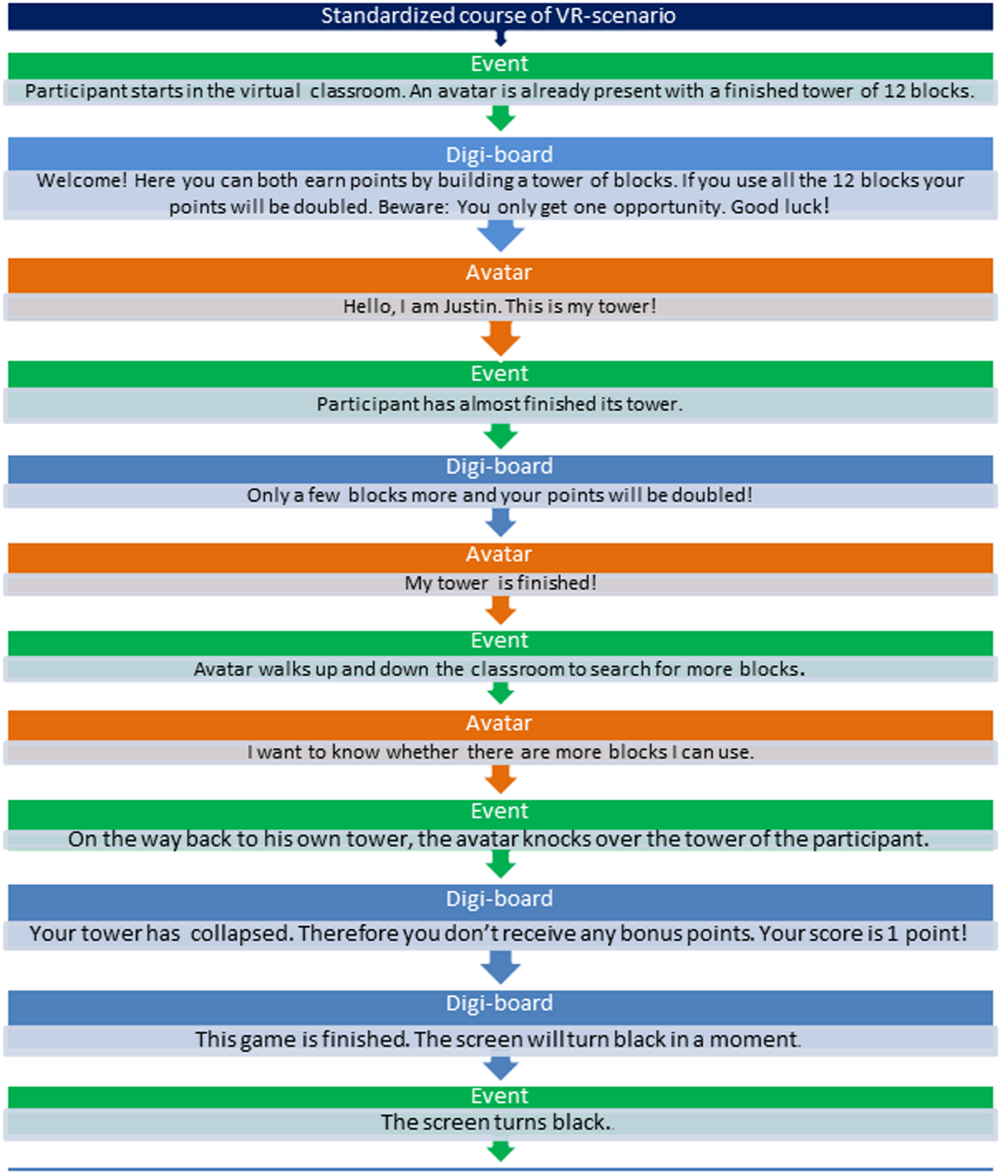
Example of a standardized course in a VR scenario for object provocation (tower game) [Colour figure can be viewed at wileyonlinelibrary.com]

Second, to promote experimental control, we scripted all social interactions between the participant and virtual peers (Figure 2). The responses of virtual peers were controlled by an experimenter, using default movements and pre‐recorded verbal responses. These standardized responses were designed to respond naturally to participants' behaviour, thus facilitating participants' immersion in the social interactions.

Third, to assess individual differences in children's aggressive SIP, we designed different social scenarios to assess both reactive and proactive aggression (Dodge, 1991). Reactive aggression is defined as an impulsive aggressive response to perceived threat or provocation, whereas proactive aggression is defined as planned aggressive behaviour aimed at obtaining a desired outcome (Dodge, 1991; Hubbard et al., 2010; Van Dijk et al., 2021). This suggests that different social contexts are needed to assess these types of aggression and their underlying SIP patterns, such as peer provocation for reactive SIP and the opportunity to obtain instrumental gain for proactive SIP. Yet most previous studies on children's SIP only used provocation scenarios, perhaps because vignettes seem less suitable to provide children with an ‘opportunity’ to aggress than to present them with a provocative event (for reviews, see Hubbard et al., 2010; Martinelli et al., 2018). As provocation scenarios seem theoretically more relevant to assess SIP underlying children's reactive aggression such as hostile intent attributions and revenge goals, earlier studies may have missed out on SIP underlying proactive aggression, such as instrumental goals (Hubbard et al., 2010). We therefore designed both provocation and instrumental gain scenarios in our VR, which we based on taxonomies of problematic situations for children with aggressive behaviour problems (Dodge et al., 1985 ***;*** Matthys et al., 2001). We used two scenarios to cover the context of provocation: being refused to join (i.e., social provocation) and participants' game being ruined (i.e., object provocation). Similarly, we used two scenarios to cover the context of instrumental gain: having the opportunity to steal (i.e., object acquisition) and having the opportunity to cheat (i.e., competition).

In sum, we designed a new interactive VR environment to assess children's aggressive SIP, aiming to accommodate for shortcomings of current assessment methods by immersing children in standardized, emotionally engaging social interactions. Therefore, in line with methodological guidelines (Boateng et al., 2018), we conducted a first‐phase pilot study to test whether our VR measure demonstrates sufficient convergent validity, measurement sensitivity and discriminant validity. We included a sample of boys recruited from both regular and special education to maximize variance in aggressive SIP. We also administered a traditional vignette‐based assessment (De Castro et al., 2005). For both VR and vignettes, we assessed children's anger, intent attributions, goals and responses. First, regarding convergent validity, we expected that the SIP assessment in VR would be positively associated with SIP assessed using vignettes. Second, regarding measurement sensitivity, we expected that the VR assessment would yield larger variances in children's SIP than the vignette assessment. Third, regarding discriminant validity, we expected that the provocation scenarios would elicit more anger, hostile intent attributions and revenge goals than instrumental gain and neutral scenarios, and more aggressive responses than neutral scenarios. We further expected that the instrumental gain scenarios would elicit more instrumental goals than the provocation and neutral scenarios, and more aggressive responses than neutral scenarios. Last, in support of potential utility of interactive VR for assessment and intervention in clinical practice, we expected that children would be more enthusiastic about participating in the VR than the vignette assessment.

## 2 METHOD

### 2.1 Participants

Thirty‐two boys ages 8 to 13 years (*M* = 10.34; *SD* = 1.36) were recruited from primary schools in the Netherlands. Children were from ethnically diverse backgrounds (34.4% Turkish/Moroccan, 15.6% Surinamese/Antillean and 50% Caucasian). To maximize variance in children's aggressive SIP, we created our sample by including boys from special education selected on aggressive behaviour problems by their teacher (*n* = 14) and a random selection of boys from regular education (*n* = 18). In special education, children were excluded if they had an autism spectrum disorder reported in their casefiles, had a clinical score on the teacher‐rated Social Responsiveness Scale (Dutch translation; Roeyers et al., 2011) or had an IQ below 80 reported in their casefiles. Parents gave written consent for their child's participation in the study. All children who received consent participated in this study (*N* = 32). This pilot study was approved by the Medical Ethics Committee of University Medical Center Utrecht.

### 2.2 Procedure

Participants were individually tested in a silent room at their school. We informed participants that the study is about peer interactions and that they would listen to stories and would enter a virtual classroom where they could interact with peers from other schools. They completed the VR‐ and vignette‐based SIP assessments on two different days with approximately a week in between (the order was counterbalanced across participants). We emphasized that no wrong answers could be given and assured participants of the confidentiality of their responses. Each assessment lasted approximately 45 min. The VR assessment was conducted by the first author (controlling the VR) and a trained graduate student (noting participants' responses). The vignette assessment was conducted by trained graduate students. At the end of each assessment and when they had completed both assessments, participants rated how enthusiastic they were about the VR and vignettes. We debriefed participants on the second assessment day, explaining that we wanted to examine how children interact with real peers rather than computer‐controlled characters. Last, they received a small gift for their participation.

### 2.3 Interactive VR

#### 2.3.1 Development

We based the content of the VR scenarios and formulation of the SIP questions on the extant literature on SIP assessment (Verhoef et al., 2019). Both were discussed in multiple feedback rounds with colleagues knowledgeable in SIP research. We conducted early try‐outs of the VR with 18 children (of the same age range as our participants) to ensure the intentions of the virtual peers were perceived as ambiguous and that the games were not too difficult but challenging enough to evoke sufficient engagement.

#### 2.3.2 VR environment

The VR environment consisted of a virtual classroom, built by CleVR (Figure 1). We introduced the classroom to participants as an actual classroom with specific behaviour rules (i.e., having respect for other children and being friendly to other children). Participants wore an HTC Vive with a combined resolution of 2160 × 1200, with an approximate diagonal field of view of 110° and support for 6DOF tracking. They could walk around freely (in a 4 × 4 space), talk with virtual peers and play games with them. Virtual peers were boys from the same age range and average height for their age. Each scenario included virtual peers that differed slightly in haircut, facial features and print of their clothing. The verbal responses of virtual peers were pre‐recorded by 12 children from theatre schools. The experimenter controlled these pre‐recorded responses, which included standardized responses used for all participants (Figure 2) and general statements allowing for a natural response to the participant (e.g., ‘I am 10. What's your age?’). During the VR scenarios, virtual peers' emotional expressions were neutral; however, when the participant aggressed, the virtual peers' expression changed to upset.

The VR environment included two games: (1) building a tower of blocks as high as possible and (2) using five balls to hit as many cans from a table as possible. We integrated high scores and bonuses in both games to increase participants' emotional engagement and to provide experimental control over gains and losses. The assessment scenarios were designed around these games, allowing participants to engage in aggression aimed at the virtual peer (e.g., hitting and name calling) as well as at the virtual peer's property (e.g., knocking over his tower). The instructions, game rules and score count were provided on a digital whiteboard and verbally explained using standard instructions recorded by a female experimenter (Figure 2).

#### 2.3.3 VR scenarios

Each scenario followed a standardized course, designed around the game played by the participant and virtual child (Figure 2). Each scenario included specific social events presented in fixed order. The exact timing of events depended on the individual participant's behaviour and progress in the game. At the end of each scenario, the experimenter presented the specific contextual event: provocation (e.g., participants' game being ruined by the virtual character) or instrumental gain (e.g., the virtual character is winning the game).

We developed six VR scenarios: one practice, one neutral, two instrumental gain and two provocation scenarios. The practice scenario served to familiarize participants with the VR environment by letting them play the game without any virtual characters. The neutral scenario served to assess participants' SIP in a situation with no engaging events (i.e., during small talk with a virtual peer). The two instrumental gain scenarios assessed SIP in response to object acquisition (i.e., participants could choose to steal a block or ball from the virtual peer to obtain additional points) and competition (i.e., participant could win the game by sabotaging the virtual peer's game). The two provocation scenarios assess SIP in response to social provocation (i.e., participants were refused to join a game by two virtual peers) and object provocation (i.e., participants' game was ruined by the virtual peer). The six VR scenarios were presented in fixed order: practice scenario, neutral scenario, object acquisition, competition, social provocation and object provocation. We expected the provocation scenarios to elicit the strongest emotions and therefore presented them last to prevent carry‐over effects.

Participants completed all six scenarios for the same game (i.e., the tower or cans game), which was randomly assigned. As such, differences in SIP between scenarios reflected scenario effects rather than game effects. A description of each scenario per type of game is provided in Table S1.

### 2.4 Measures

#### 2.4.1 SIP assessment using VR

We assessed participants' aggressive responding by observing their actual behavioural response in the VR scenario. We assessed their SIP by asking several questions directly after each scenario. During these 1‐min assessments between scenarios, participants kept the VR glasses on. We assessed participants' anger, intent attributions, interaction goals, aggressive response generation and evaluation of their actual aggressive response in VR. For the present study, however, we excluded aggressive response generation because too few children mentioned additional aggressive responses in addition to their actual response, and we excluded evaluation of aggression because we could only assess this variable if children responded aggressively, resulting in too few observations to analyse.

##### Anger

Participants' anger in VR was assessed with one item: ‘The other boy did [behavior other boy]. How angry did this make you feel, on a scale from 1–10?’. Anger scores were averaged, creating separate scores for provocation (two items; *r* = .78), instrumental gain (two items; *r* = .53) and neutral contexts (one item).

##### Hostile intent attribution

Participants' intent attributions were assessed using two items following each VR scenario: ‘The other boy did [behavior other boy]. To what extent did he try to be mean, on a scale from 1–10?’ and ‘To what extent did he try to hinder you, on a scale from 1–10?’. Correlations between the two items within each VR scenario were acceptable (*M* = .73, *Mdn* = .77, range = .34–.91) and therefore averaged to create a single hostile intent attribution score for each scenario. Next, hostile intent attribution scores were averaged, creating separate scores for provocation (two items; *r* = .53), instrumental gain (two items; *r* = .64) and neutral contexts (one item).

##### Goals

Participants' goals were assessed using one open‐ended question following each VR scenario: ‘When the other boy did [behavior other boy], you did [behavior participant]. What was the reason you did this?’. During the assessment, a trained graduate student directly wrote down all participants' answers. Afterwards, we coded these responses in line with previous research (De Castro et al., 2012) into the categories *no goals* (e.g., ‘I don't know’ and ‘I had no goal’), *revenge goals* (e.g., ‘to retaliate’ and ‘because I was angry’), *instrumental goals* (e.g., ‘to win the game’ and ‘to show him who's boss’) and *goals underlying non‐aggressive behaviour* (e.g., ‘to become friends’ and ‘to avoid problems’). A second rater also coded 50% of the transcriptions. Inter‐rater reliability was good, with *κ* ranging from 0.88 to 1.00 (*M* = .93, *Mdn* = .88). Scores for revenge goals were created by assigning 1 to *revenge goals* codes and 0 to other codes and then averaged to create separate scores for provocation (two items; *τ* = .53), instrumental gain (two items; *τ* = .56) and neutral contexts (one item). Similarly, scores for instrumental goals were created by assigning 1 to *instrumental goals* codes and 0 to other codes and averaged to create separate scores for provocation (two items; *τ* = .47), instrumental gain (two items; *τ* = .68) and neutral contexts (one item).

##### Behavioural responses

Behavioural responses in VR were assessed through observation of participants' behaviour during each scenario. A trained graduate student directly wrote down participants' behaviour. We coded this behaviour afterwards using standard procedures (De Castro et al., 2005) into the categories 0 for *non‐aggressive behaviour* (e.g., prosocial and avoidance), 1 for *mild aggressive behaviour* (e.g., coercion and verbal aggression) and 2 for *severe aggressive behaviour* (e.g., physical aggression and destructive aggression). A second rater also coded 50% of the behavioural descriptions. Inter‐rater reliability was good, with *κ* ranging from 0.87 to 1.00 (*M* = .97, *Mdn* = 1.00). Aggressive response scores were averaged, creating separate scores for provocation (two items; *r* = .55), instrumental gain (two items; *r* = .92) and neutral contexts (one item).

#### 2.4.2 SIP assessment using vignettes

We used a validated vignette measure to assess participants' SIP (De Castro et al., 2005). This measure—as most standard SIP measures—only includes provocation stories. Participants were presented with five audiotaped vignettes describing ambiguous peer provocations, such as losing a computer game through fault of a peer (De Castro et al., 2005). We informed participants that they would listen to vignettes about daily social events and asked them to imagine each story was actually happening to them. Participants first practised with one vignette, so that the experimenter could check whether they understood the procedure (all participants did). Next, following each vignette, we assessed children's SIP using the same questions and coding schemes as used for the VR assessment, except for two minor modifications. First, we formulated SIP vignette questions as hypothetical (‘What would you …?’) instead of actual (‘What did you …?’). Second, we assessed aggressive responding using an open‐ended question (i.e., ‘What would you do if [peer provocation]?’) instead of observation. Hostile intent attribution items were correlated within each vignette and therefore averaged (*M* = .76, *Mdn* = .80, range = .66–.83). Inter‐rater reliability (*κ*) was based on 50% of transcriptions and was acceptable for both interaction goals (*M* = .80, *Mdn* = .77, range = .66–1.00) and aggressive responding (*M* = .79, *Mdn* = .78, range = .77–.86). We averaged participants' responses across the five vignettes, creating single scores for anger (*α* = .61), hostile intent attribution (*α* = .72), revenge goals (*α* = .78) and aggressive responding (*α* = .74).

#### 2.4.3 Enthusiasm about the VR and vignette assessments

We assessed children's enthusiasm about the VR and vignette assessments using five items at the end of each assessment (e.g., ‘How much did you like the VR/vignettes?’ and ‘How much would you like to do the VR/vignettes again?’). Children responded on a rating scale from 1 (*not at all*) to 10 (*very much*). We averaged across the five items to create enthusiasm scores for both VR (*α* = .87) and vignettes (*α* = .86). To capture children's explicit comparison, we also asked them to rate how much they liked the VR and vignette assessments on a scale from 1 to 10 after they had completed both assessments.

### 2.5 Statistical analyses

We had four main goals. First, we examined the convergent validity of SIP assessment in VR by calculating correlations between VR‐ and vignette‐assessed SIP variables, using Pearson's *r* and Kendall's *τ*. In these analyses, we only included the VR provocation scenarios to relate to the vignette scores, because the vignettes only covered the domain of provocation. We analysed correlations for anger, hostile intent attribution, revenge goals and aggressive responding (i.e., the SIP variables relevant to provocation contexts). Second, we examined measurement sensitivity by comparing the variances of VR‐ and vignette‐assessed SIP variables (i.e., anger, hostile intent attribution, revenge goals and aggressive responding). To this end, we used the Pitman–Morgan test based on Spearman's rank correlations (McCulloch, 1987). Third, to test the discriminant validity of SIP assessment in VR, we conducted planned comparisons of participants' SIP between provocation, instrumental gain and neutral contexts, using paired *t*‐tests. Fourth, we examined whether children were more enthusiastic about our SIP assessment in VR than with vignettes, using paired *t*‐tests. Given the small sample size and non‐normal distribution of the variables, we conducted these analyses using bootstrapped bias‐corrected accelerated (BCa) 95% confidence intervals (CIs) based on 5000 resamples.

## 3 RESULTS

### 3.1 Convergent validity: Association between SIP in VR versus vignettes

Table 1 presents the zero‐order correlations between VR‐ and vignette‐assessed SIP variables. CIs that exclude the value of 0 signify that the correlation was significant. Supporting convergent validity, we found small to high significant correlations between VR‐ and vignette‐assessed anger (*r* = .37, BCa 95% CI: .02–.65), hostile intent attribution (*r* = .56, BCa 95% CI: .28–.79), revenge goals (*τ* = .67, BCa 95% CI: .46–.84) and aggressive responding (*r* = .73, BCa 95% CI: .50–.89). These results indicate that children's SIP assessed with interactive VR corresponds with their SIP assessed through a traditional validated vignette‐based measure.

**TABLE 1:** Bivariate correlations between SIP variables in provocation VR contexts and vignettes

	Range	*M* (*SD*)	2.	3.	4.	5.	6.	7.	8.
1. VR: Anger	2.50–10.00	7.09 (2.60)	.73*	.48*	.49*	.37*	.49*	.53*	.58*
2. VR: Hostile intent attribution	1.25–10.00	6.76 (2.54)		.41*	.52*	.13	.56*	.38*	.47*
3. VR: Revenge goals	0.00–1.00	0.38 (0.42)			.87*	.21	.25	.67*	.72*
4. VR: Aggressive responding	0.00–2.00	0.88 (0.87)				.35*	.37*	.60*	.73*
5. Vignette: Anger	2.80–10.00	6.88 (1.86)					.34*	.37*	.48*
6. Vignette: Hostile intent attribution	1.00–8.90	3.72 (1.99)						.35*	.45*
7. Vignette: Revenge goals	0.00–1.00	0.28 (0.33)							.93*
8. Vignette: Aggressive responding	0.00–1.80	0.45 (0.53)							

*Note*: All correlations including revenge goals are calculated using Kendall's *τ*, and other correlations used Pearson's *r*.

* Indicates significance at .05, as the bootstrap 95% confidence interval did not include zero.

### 3.2 Measurement sensitivity: Variances of SIP in VR versus vignettes

To examine whether VR captured more individual differences in SIP than the vignettes, we compared variances of SIP variables between VR and vignettes. Table 1 shows the standard deviations for VR‐ versus vignette‐assessed SIP variables, with larger standard deviations signifying larger variances. Results revealed significantly larger variances in SIP using VR versus vignettes for aggressive responding, *t*(30) = 4.09, *p* < .001, but not for anger, *t*(30) = 1.43, *p* = .163, hostile intent attribution, *t*(30) = 1.40, *p* = .173, and revenge goals, *t*(30) = 2.01, *p* = .053. So, only for aggressive responding, we found larger variances, meaning that interactive VR is more sensitive to capture individual differences in children's aggressive responding compared to vignettes.

### 3.3 Discriminant validity: SIP outcomes across VR contexts

Table 2 presents the descriptive statistics of the VR‐assessed SIP variables for the provocation, instrumental gain and neutral contexts separately, as well as the significance levels for our planned comparisons. Supporting discriminant validity, we found that the provocation context elicited significantly more anger (*d* = 1.56), hostile intent attributions (*d* = 1.62) and revenge goals (*d* = 0.83) than the instrumental gain context. As predicted, it also elicited significantly more anger (*d* = 1.89), hostile intent attributions (*d* = 1.89), revenge goals (*d* = 0.71) and aggressive responses (*d* = 0.84) than the neutral context. However, contrary to expectations, the instrumental gain context did not elicit significantly more instrumental goals than the provocation context (*d* = 0.31) or the neutral context (*d* = 0.29), nor did it elicit more aggressive responses than the neutral context (*d* = 0.36). Taken together, these findings provide partial support for the use of distinct VR contexts to capture distinct SIP patterns in children.

**TABLE 2:** Descriptive statistics of SIP variables per context and significance levels for planned comparisons between contexts based on bootstrap 95% confidence intervals

	Provocation context	Instrumental gain context	Neutral context	Provocation versus neutral	Provocation versus instrumental	Instrumental versus neutral
	Mean (*SD*)	Mean (*SD*)	Mean (*SD*)	*p*	*p*	*p*
Anger	7.09 (2.60)	3.13 (2.80)	1.81 (2.11)	<.001	<.001	
Hostile intent attribution	6.76 (2.54)	2.48 (2.43)	2.05 (1.86)	<.001	<.001	
Revenge goals	0.38 (0.42)	0.06 (0.21)	0.03 (0.18)	<.001	.001	
Instrumental gain goals	0.06 (0.21)	0.17 (0.35)	0.06 (0.25)		.113	.114
Aggressive responding	0.88 (0.87)	0.47 (0.84)	0.19 (0.59)	.001		.068

*Note*: Cells are empty if we had no hypotheses about this comparison.

### 3.4 Enthusiasm about the VR and vignette assessments

We asked children to rate their enthusiasm directly after each assessment and after completing both assessments. As predicted, children were more enthusiastic about the VR assessment (*M* = 8.54, *SD* = 1.98) than the vignette assessment directly after completing each assessment (*M* = 6.94, *SD* = 2.17) directly after each assessment, *p* = .001, *d* = 0.72. They also gave higher ratings to the VR (*M* = 9.06, *SD* = 1.95) than the vignettes (*M* = 6.78, *SD* = 2.56) after completing both assessments, *p* < .001, *d* = 1.08.

## 4 DISCUSSION

This pilot study examined whether interactive VR provides a valid assessment of children's aggressive SIP. We developed a virtual classroom where children played games with virtual peers. Children reported on their SIP in three distinct VR contexts (i.e., neutral, instrumental gain and provocation) and also completed a vignette‐based assessment of SIP. Supporting convergent validity, results showed positive associations between VR‐ and vignette‐based assessments of children's anger, hostile intent attributions, revenge goals and aggressive responding. Supporting measurement sensitivity, results showed larger variances of SIP assessment in VR for aggressive responding in VR versus vignettes. However, variances did not differ between VR and vignettes for anger, hostile intent attributions and revenge goals. Supporting discriminant validity, results showed that the provocation context elicited more anger, hostile intent attributions and revenge goals than the instrumental gain and neutral contexts, and more aggressive responses than the neutral context. However, one aspect of discriminant validity was not supported: the instrumental gain context did not elicit more instrumental goals than provocation and neutral contexts, nor more aggressive responses than the neutral context. Last but not least, results showed that children were more enthusiastic about participating in VR than completing a vignette‐based measure of SIP.

Several findings stand out, warranting further discussion. We found significant correlations between SIP in VR and vignettes, supporting the convergent validity of our new measure. Yet, although correlations were moderate to large for hostile intent attributions, revenge goals and aggressive responses, they were small for anger. This may be caused by the potentially limited reliability of using vignettes to assess children's anger. Participants may have found it difficult to report on their anger in hypothetical scenarios—in fact, people generally struggle to report on anticipated negative affective states (for a review, see Robinson & Clore, 2002). Perhaps, interactive VR could provide a more reliable assessment of children's anger than current instruments do—a direction for future research worth investigating.

Another notable finding is that the improved measurement sensitivity of our interactive VR assessment was supported for aggressive responding, but not for other SIP variables. This could be due to the small sample size of our pilot study: variances were larger in VR for all SIP variables, but these differences were not significant. Still, the larger variance for aggressive responding in VR suggests that VR may be more sensitive to assess individual differences in aggressive responding than current vignette‐based methods. As interactive VR immerses children in actual social interactions and allows them to actually aggress against virtual peers—an important difference with vignettes, which ask children to reflect on their hypothetical aggressive responses—it may have triggered aggressive responses in some children that were not triggered by vignettes. This notion aligns with theoretical work suggesting that many children may only respond aggressively when they are emotionally engaged (Anderson & Bushman, 2002; Lemerise & Arsenio, 2000). For clinical practice, this finding suggests that interactive VR assessment may detect individual differences in children's aggressive responding that would remain undetected when using measures that ask children to reflect on their hypothetical responses.

We developed distinct VR scenarios, aiming to assess the distinct SIP patterns that may underlie children's reactive and proactive aggression (Hubbard et al., 2010). Our findings showed that VR scenarios including peer provocation elicited more anger, hostile intent attributions and revenge goals than the instrumental gain and neutral contexts, and more aggressive responses than the neutral context. These findings align with social‐cognitive theory suggesting that contexts where the participant is provoked, frustrated or threatened should evoke SIP patterns related to reactive aggression (Dodge, 1991). They also align with empirical research suggesting that children's aggressive SIP patterns are context dependent, as we found that the same children showed aggressive SIP in some VR scenarios but not others (De Castro & Van Dijk, 2017). These findings underscore the relevance of using distinct contexts to validly assess children's aggressive SIP patterns—and interactive VR may be an engaging and flexible method to do so.

Yet what stood out is that our instrumental gain scenarios—developed to assess SIP underlying proactive aggression—did not elicit more instrumental goals than the provocation or neutral contexts. An explanation for this finding could be that proactive aggression is relatively rare (Dodge et al., 1997; Thomson & Centifanti, 2018). Indeed, few children in our sample displayed instrumental SIP in our VR scenarios, reducing statistical power to find significant differences between contexts. This idea is reflected in our data: although non‐significant, on average, children showed more instrumental goals in the instrumental gain versus other contexts. Research in a larger sample of children is needed to examine whether instrumental gain contexts indeed elicit more instrumental tendencies than other contexts. This would be worthwhile, because VR—due to its realistic nature—seems more suitable to present instrumental gain scenarios than hypothetical vignettes (in fact, vignette‐based SIP assessments have rarely included instrumental gain scenarios).

Lastly, children reported to be more enthusiastic about participating in VR than completing a vignette‐based measure of SIP. This finding may have important implications for clinical practice. VR could be an attractive option for psychological assessment and intervention, because it may increase children's motivation to participate. If future research supports this idea, this may be particularly relevant for children with aggressive behaviour problems because they are often less motivated to engage in therapy (Frick, 2012).

This study had several strengths. To our knowledge, it is the first study that used interactive VR to assess children's aggressive SIP. Moreover, we maximized variance in children's SIP by recruiting children from both regular and special education for children with behaviour problems. The use of interactive VR in a sample with substantial variance in SIP allowed us to examine individual differences in children's aggressive SIP in an ecologically valid, experimentally controlled, theoretically comprehensive and engaging context.

This study also had several limitations. First, the small sample size of this pilot study limits generalization of the findings. Moreover, it prevented us from running additional analyses, for instance, to test whether the order in which children completed the VR‐ and vignette‐based assessments, despite counterbalancing, may have affected the results. Second, the provocation and instrumental gain contexts were assessed using only two VR scenarios each, reducing reliability of SIP measurements within each context. Relatedly, since research demonstrated that children display aggression in different contexts (e.g., Matthys et al., 2001), using two scenarios for each context may not have covered the broad range of social situations known to evoke aggression in children. Third, we only ran convergent validity analyses for the provocation VR scenarios because—to our knowledge—no well‐established instrumental gain vignettes exist. Fourth, although the interactive nature of VR might have enhanced children's engagement in the actual interactions, it also caused the VR scenarios to slightly differ between children.

The findings of this pilot study open up valuable opportunities for both research and clinical practice. For research, an important next step is to examine to what extent SIP and behaviour assessed in VR predict real‐life aggression, using parent‐ or teacher‐report questionnaires, peer nomination and observation (Boateng et al., 2018). Moreover, the greater experimental control over social stimuli provided in the VR environment (e.g., non‐verbal behaviours and emotion expressions) will allow researchers to test more specific hypotheses about causal effects in children's social interactions. For clinical practice, interactive VR—if it is further validated by future research—may provide a more attractive, flexible and valid method to assess children's aggressive SIP. Ultimately, interactive VR may also provide inroads for intervention. Clinicians could use the flexible VR environment to create engaging exercises tailored to individual clients, with precise control to adapt difficulty and complexity during the intervention.

In sum, this pilot study suggests that interactive VR is a promising tool to assess children's aggressive SIP. The use of VR allows researchers and clinicians to assess aggressive SIP in an emotionally engaging, ecologically valid context that is interactive and realistic. In the future, VR may further our understanding of SIP patterns underlying children's aggression and be used to enhance assessment and intervention for children with aggressive behaviour problems.

## CONFLICT OF INTEREST

We have no known conflict of interest to disclose.

## AUTHOR CONTRIBUTIONS

The study was designed by all authors. Material preparation was performed by all authors. Data collection was performed by Rogier E. J. Verhoef and trained graduate students. Analyses were performed by Rogier E. J. Verhoef. The first draft of the manuscript was written by Rogier E. J. Verhoef and edited by all authors. All authors commented on previous versions of the manuscript. All authors read and approved the final manuscript.

## ETHICS APPROVAL

The study was approved by the Medical Ethics Committee of University Medical Center Utrecht and conducted in accordance with the 2013 Helsinki Declaration.

## CONSENT TO PARTICIPATE

Written informed consent for the study was obtained from parents.

## Supporting information

**Table S1.** Description of VR‐Scenarios per Game; Italics Indicate Differences Between Games.

## Data Availability

The data that support the findings of this study are available through the Open Science Framework at https://doi.org/10.17605/OSF.IO/CS3AK. The syntax of the analyses run for this study are available through the Open Science Framework (see link above).
