# Supplementary material for: Interactive virtual reality assessment of aggressive social information processing in boys with behaviour problems: A pilot study
Source: Clin Psychol Psychother. 2021 Jun 17;28(3):489–99. doi: 10.1002/cpp.2620 (PMC8361679; doi:10.1002/cpp.2620)
Supplement: Supplementary file 1 — Table S1. Description of VR‐Scenarios per Game; Italics Indicate Differences Between Games. [file CPP-28-489-s001.docx]

**Supplementary Materials**

**Table S1**

*Description of VR-Scenarios per Game; Italics Indicate Differences Between Games.*

| VR-scenario | Tower game | Cans game |
| --- | --- | --- |
| Practice | The behavioral rules in the classroom are explained and the participant practices the game. The participant chats with a computerized avatar to practice talking in VR. | The behavioral rules in the classroom are explained and the participant practices the game. The participant chats with a computerized avatar to practice talking in VR. |
| Neutral | The participant *builds a tower of blocks*. During the game a virtual child engages in small-talk with the participant. | The participant *throws cans from the table*. During the game a virtual child engages in small-talk with the participant. |
| Object acquisition | The participant *builds a tower of blocks as high* as possible, however is one *block* short to finish the *tower* and earn bonus-points. During the game a virtual child is also *building a tower*, but then leaves the room and asks the participant if he could watch *his tower and blocks* until he returns. | The participant *throws as much cans from a table* as possible, however is one *ball* short to finish the *game* and earn bonus-points. During the game a virtual child is also *throwing cans from a table*, but then leaves the room and asks the participant if he could watch *his cans and balls* until he returns. |
| Competition | The participant and a virtual child both *build a tower of blocks as high* as possible. They are instructed that the player with the *highest tower* earns bonus-points, and that none of the players get bonus-points when it turns out to be a draw. The virtual child *has finished his tower* before the participant does, and announces that he is winning. | The participant and a virtual child both *throw as much cans from a table* as possible. They are instructed that the player with the *most cans thrown from the table* earns bonus-points, and that none of the players get bonus-points when it turns out to be a draw. The virtual child *has almost thrown all his cans from the table* before the participant does, and announces that he is winning. |
| Social provocation | The participant sees two virtual children in the classroom who are busy *building a tower of blocks*. The participant is prompted by the digital whiteboard to ask if he can join the game. Upon asking this question, the virtual children tell the participant he cannot join the game. | The participant sees two virtual children in the classroom who are busy *throwing cans from a table*. The participant is prompted by the digital whiteboard to ask if he can join the game. Upon asking this question, the virtual children tell the participant he cannot join the game. |
| Object provocation | The participant *builds a tower of blocks as high* as possible. A virtual child is already present with *a finished tower*. When the participant has *finished his tower*, the virtual child *walks up and down the classroom in search of more blocks. On his way back, the virtual child knocks over the tower*, thus ruining the participant’s game. | The participant *throws as much cans from a table* as possible. A virtual child is already present with *his own balls*. When the participant has *one ball left to throw*, the virtual child *picks up the participant’s last ball and drops it. The ball rolls out of reach,* thus ruining the participant’s game. |

*Note*. In all scenarios participants can earn points by playing the game: 1 point for each block built or each can thrown over, and bonus points for completing the game.
